# Comparing visual and automated urine dipstick analysis in a general practice population

**DOI:** 10.1080/02813432.2024.2392776

**Published:** 2024-08-20

**Authors:** S. M. L. Cox, P. Hoitinga, G. J. Oudhuis, R. M. Hopstaken, P. H. M. Savelkoul, J. W. L. Cals, E. G. P. M. de Bont

**Affiliations:** a Department of Family Medicine, CAPHRI, Maastricht University, Maastricht, The Netherlands; b Medical Microbiology, Infectious Diseases, and Infection Prevention, Maastricht University Medical Centre+, Maastricht, The Netherlands; c GP Practice De Kuil, Hapert, The Netherlands

**Keywords:** Urinary tract infections, point-of-care testing, general practice, primary health care, diagnostic equipment

## Abstract

**Introduction:**

Urinary symptoms constitute the primary reason for female patients to consult their general practitioner. The urinary dipstick test serves as a cornerstone for diagnosing urinary tract infections (UTIs), yet traditional visual interpretation may be subject to variability. Automated devices for dipstick urinalysis are routinely used as alternatives, yet the evidence regarding their accuracy remains limited. Therefore we aimed to compare concordance between visual and automated urinary dipstick interpretation and determine their test characteristics for the prediction of bacteriuria.

**Material and methods:**

We conducted a prospective validation study including urine samples originating from adult patients in general practice that were sent to the Maastricht Medical Centre + for urinary culture. Urinary dipstick tests were performed on each sample, which were interpreted visually and automatically. We calculated Cohen’s κ and percentage agreement and used 2 × 2 tables to calculate test characteristics.

**Results:**

We included 302 urine samples. Visual and automated analysis showed almost perfect agreement (κ = 0.82 and κ = 0.86, respectively) for both nitrite and leukocyte esterase, but moderate agreement for erythrocytes (κ = 0.51). Interpretation of clinically relevant (nitrite and/or leukocyte esterase positive) samples showed almost perfect agreement (κ = 0.88). Urinary dipsticks show similar test characteristics with urinary culture as gold standard, with sensitivities of 0.92 and 0.91 and specificities of 0.37 and 0.41 for visual and automated interpretation respectively.

**Conclusion:**

Automated and visual dipstick analysis show near perfect agreement and perform similarly in predicting bacteriuria. However, automated analysis requires maintenance and occasionally measurement errors can occur.

## Introduction

Urinary tract complaints are the most common cause for patients to visit their general practitioner (GP). In 2022, 148.5 out of every 1000 general practice consultations in the Netherlands took place because of urinary tract infections (UTIs) [1]. Like in Denmark, Dutch general practitioners diagnose a UTI based on patient symptoms combined with the urinary dipstick test, possibly followed by microbiological analysis at a specialised laboratory [2,3]. In the Netherlands, there is the extra option of a dip slide incubation of the patient’s urine, but dip slides are not accepted by every laboratory for further analysis [2]. Nonetheless, the dipstick test determines the presence of leukocyte esterase and nitrite in urine, which indicate the presence of inflammation and bacteria respectively. Incorrectly analysed dipstick results may lead to overprescription of antibiotics [4]. It is estimated that at least one-third of antibiotic prescriptions are inappropriate, leading to adverse effects and facilitating the rise of antibiotic resistance [5,6].

Visual interpretation of urinary dipsticks is potentially subject to a variety of errors [7,8]. Developers sought to diminish the frequency of these errors with automated dipstick testing. Automated testing makes use of reflectance photometry to interpret the colour change on the dipstick [9]. This would take the burden of interpretation away from the clinician, resulting in a more objective evaluation of results [10,11]. Previous research has shown that point-of-care dipstick urinalysers are able to detect nitrite as well as a standard laboratory test (Urisys 2400, Roche), while leukocyte esterase and erythrocyte were detected with lower sensitivity [9]. Furthermore, agreement with the laboratory standard test was similar for automated and visual interpretation of nitrite, leukocyte esterase and erythrocyte results [12].

Although automated dipstick interpretation has demonstrated parity with the laboratory standard (particularly in interpreting urinary nitrite), Dutch, Danish, Norwegian and Swedish standards for GPs currently omit this laboratory test for UTI diagnosis. Instead, when further diagnostics are warranted, GPs rely on submitting urine samples from suspected UTI patients for urinary culture with antibiotic susceptibility testing [2,3,13,14]. Existing studies do not address potential disparities in outcomes within the decision algorithm outlined in the Dutch guidelines when employing visual versus automated urinalysis. This study seeks to explore the concordance between visual and automated urinary dipstick interpretation and determine their test characteristics for the prediction of bacteriuria.

## Materials and methods

### Study setting

We conducted a prospective validation study, comparing visual and automated interpretation of urinary dipstick tests. We included urinary samples sent in from general practices (both in and out-of-hours) for urinary culture between January and March 2023. The samples underwent regular diagnostics (Gram staining and urinary culture), before we performed the urinary dipstick test. Results of the urinary culture served as gold standard for determining the sensitivity, specificity, positive predictive value and negative predictive value of the dipstick test.

### Inclusion criteria

We included non-catheter urinary samples sent in for urinary culture originating from general practice. We excluded samples originating from patients younger than 18 years of age, since UTI manifests itself differently in children [15].

### Urinary culture

A 1 µL inoculation loop was used to inoculate a blood agar plate (Becton Dickinson, Franklin Lakes, New Jersey, United States) containing 5% sheep blood and a ­cystine–lactose–electrolyte deficient (CLED) agar plate (Becton Dickinson, Franklin Lakes, New Jersey, United States). The plates were aerobically incubated (C 150, Binder GmbH, Tuttlingen, Germany) at 35 degrees Celsius for 24 h. If growth had not developed within the first 24 h, the agars were cultured in the same conditions for another 24 h. If growth was observed, pathogen identification took place using matrix assisted laser desorption/ionization time-of-flight analysis (MALDI-TOF) (VITEK MS^™^, bioMérieux, Marcy-l’Étoile, France).

### Urinary dipstick test

We dipped Combur^10^ Test UX strips (Roche Diagnostics, Basel, Switzerland, 65821903) in homogenised urine for one second until all test fields had made contact with the sample. Subsequently we transferred the test strip to the Roche Urisys 1100 device (Roche Diagnostics, Basel, Switzerland, 05203281), which uses reflectance photometry to interpret the colour change on the dipstick. We chose this device since it performed the best in previous research [9]. After automated urinalysis, the test strip was read visually by SMLC (biomedical researcher) and PH (medical student), the interpreters being blinded for the result of the automated analysis. Nitrite, leukocyte esterase and erythrocyte fields were compared to the legend on the bottle. Possible results were ‘negative’ and ‘positive’ for nitrite; ‘negative’, ‘1+’, ‘2+’ and ‘3+’ for leukocyte esterase; and ‘negative’, ‘1+’, ‘2+’, ‘3+’ and ‘4+’ for erythrocytes. If interpreters’ results differed, consensus was reached before recording of the result. Quality control of the device was performed weekly via Control-Test M strips (Roche Diagnostics, Basel, Switzerland (10)10088825).

### Data analysis

Since Dutch guidelines only distinguish between positive and negative leukocyte esterase and erythrocyte dipstick tests, results were dichotomised to negative (when the result was negative) and positive (when the result was 1+ or higher). Agreement of both interpretation methods was determined using percentage agreement and Cohen’s κ. We determined the test characteristics of the dipstick test using 2 × 2 tables, comparing test results to urinary culture results. We conducted the statistical analyses using IBM SPSS Statistics for Windows (Version 28.0. Armonk, NY: IBM Corp).

## Results

### Patient characteristics

We measured 307 samples, of which we had to exclude five (1.6%) samples due to measurement errors. Therefore, we included 302 patient samples, of which 205 (67.9%) originated from female patients. The median patient age was 69 years (range 19–98). Roughly half (52.6%) of all included samples were tested positive for bacteriuria with urinary culture.

### Agreement of visual and automated dipstick interpretation

Table 1 shows the agreement between the dipstick results as rated by visual and automated interpretation. The percentage of test results that were rated the same between tests was >90% for the nitrite and leukocyte tests, but was lower for the erythrocyte test. Cohen’s κ shows almost perfect agreement between visual and automated interpretation for nitrite and leukocyte esterase. Full contingency tables are available in Appendices 1–3.

**Table 1.** Agreement of visual and automated interpretation of urinary dipsticks.

	Percent agreement	Cohen’s κ (95% CI)
Nitrite	92.1%	0.82 (0.81–0.83)
Leukocyte esterase	94.7%	0.86 (0.80–0.91)
Erythrocytes	83.4%	0.51 (0.43–0.58)

CI, confidence interval.

### Clinical evaluation

The decision algorithms of the Dutch and Danish UTI guidelines consider nitrite and/or leukocyte esterase positive urine as potentially constituting a UTI [2]. Table 2 shows the agreement of visual and automated urinary dipstick results in determining potential UTIs. The methods show almost perfect agreement.

**Table 2.** Agreement of visual and automated interpretation on determining clinically relevant samples.

Automated visual	Negative	Positive
Negative	64	4
Positive	9	225
Cohen’s κ (95% CI)	0.88 (0.82–0.94)	

CI, confidence interval.

To determine whether automated interpretation would lead to a different diagnosis compared to visual interpretation, we compared results of both methods to the result of the urinary culture. Table 3 shows that both methods have >90% sensitivity, but a specificity <50%. Test characteristics did not differ significantly between methods. Additionally, Appendices 4 and 5 show high specificity and low sensitivity for only the nitrite test for both interpretation methods, and vice versa for only the leukocyte esterase test. Again, there is no significant difference in test characteristics between the interpretation methods.

**Table 3.** Test characteristics of visual and automated interpretation with urinary culture as gold standard.

Culture visual	Negative	Positive	Culture automated	Negative	Positive
Negative	53	15	Negative	59	14
Positive*	90	144	Positive*	84	145
Test characteristics visual		95%CI		Test characteristics automated	95% CI
Sensitivity	0.91	0.86–0.95	Sensitivity	0.91	0.87–0.96
Specificity	0.37	0.29–0.45	Specificity	0.41	0.33–0.49
PPV	0.62	0.55–0.68	PPV	0.63	0.57–0.70
NPV	0.78	0.60–0.71	NPV	0.81	0.72–0.90

*Positive for nitrite and/or leukocyte esterase.

CI, confidence interval; NPV, negative predictive value; PPV, positive predictive value.

## Discussion

### Summary

This study sought to explore the concordance between visual and automated urinary dipstick interpretation and determine their test characteristics for the prediction of bacteriuria. Visual and automated interpretation of urinary dipstick results show almost perfect agreement for the nitrite and leukocyte esterase test. However, the two methods agree only moderately for interpretation of the erythrocyte result. When looking at the clinically relevant outcomes as described in the Dutch and Danish guidelines, the two methods show almost perfect agreement. Test characteristics of the two methods do not differ significantly, with high sensitivity but poor specificity.

### Strengths and limitations

This study was a prospective validation study in which we were able to systematically collect many samples in a short time-period. We compared the tests to the current gold standard and the point-of-care analyser has been previously evaluated and validated in routine general practice [9, 12]. Furthermore, the tests were performed on clinically relevant samples, representing the general population of samples that was sent in for urinary culture.

This study also has its limitations. The visual interpreters of the dipstick test were not general practice nurses or general practitioners. Furthermore, we have no records of the individual observers; therefore we were unable to determine inter-observer reliability. Additionally, results obtained from urine samples sent in for culture are not generalisable to all samples in general practice, since Dutch guidelines suggest only ordering urinary culture in case of high-risk patients [2]. High-risk patients include frail elderly patients with therapy failure, which might explain the relatively high age of the patients included in this study. Previous studies showed average ages of 43–54 years for patients consulting their GP with urinary symptoms, considerably lower than the average age in our study [4, 16–18]. Furthermore, since we did not have access to the sample until arrival at the hospital (and therefore were not able to speak to the patients), we were unaware of any symptoms they might have had. Therefore, it was impossible for us to differentiate between asymptomatic bacteriuria and actual urinary tract infections. A patient has asymptomatic bacteriuria when bacteria are present in their urine without causing symptoms typically associated with UTI. Asymptomatic patients do not need to be treated, but since their diagnostic results show bacteria to be present in their urine they often still are. This results in overprescription of antibiotics, leading to antimicrobial resistance [19]. Asymptomatic bacteriuria is more prevalent with age and might therefore be overrepresented in our population [20]. However, since Dutch guidelines suggest urinary culture only in high-risk patients (patients with recurrent UTI, vulnerable elderly, diabetic patients, patients with signs of tissue invasion, such as fever, shivers, signs of sepsis, etc.) we assume that culture positive cases in our study have a high likelihood of being an actual UTI. Additionally, due to our inclusion method we have no records of the time and manner of sample voiding. Storage details at the general practices were also unknown. We suggest repeating this study in daily general practice with records of patient symptoms in order to draw definitive conclusions.

### Comparison with existing literature

In this study we show a direct comparison between visual and automated interpretation of the urinary dipstick. We see high agreement between the two methods for the nitrite and leukocyte esterase test, but only moderate agreement for the erythrocyte test. These findings corroborate the results found by Schot et al. who show similar agreements between the Urisys 1100 and the laboratory standard test [9]. The low agreement for erythrocytes seen in our study is mainly caused by samples that were scored negative during visual interpretation, while scoring positive during automated interpretation. We think this has mainly to do with the legend on the tube of the Combur^10^ Test UX strips, since there it shows ‘1+’ as several green spots on a yellow background, while the automated analysis already scored samples positive when only 1 or 2 spots were visible. This disparity is not as relevant for Dutch GPs, since, like in Denmark, erythrocytes are omitted from the decision algorithm [2,3].

Furthermore, we show that there is no significant difference between visual and automated interpretation of urinary dipstick results for urinary tract infections in terms of clinically relevant samples. This only rings true if the dipstick test is performed as instructed by the manufacturer and is read within 2 min. Though easy to use, the Urisys 1100 needs to be calibrated weekly and cleaned properly after every sample to prevent unreliable results [21]. False positives in the automated dipstick analyses may be due to the discolouration of the dipstick, caused by haematuria, concentrated urine, or smudging from adjacent test fields. On five separate occasions an error occurred during automated interpretation, which we suspect was due to the sample being highly positive for nitrite. In these cases reagent from the nitrite field would spill over to adjacent fields, contaminating the signal in those fields in the process. Visually the samples were still able to be interpreted, but since the machine is calibrated for certain wavelengths in specific fields, it will cause an error when a different wavelength is measured.

### Implications for research and/or practice

The test characteristics of the urinary dipstick is similar for both methods when following the decision algorithm of the Dutch and Danish guidelines [2,3]. According to these guidelines, a patient whose urine tests positive for nitrite (when patient symptoms make a UTI plausible), is to be diagnosed with a UTI. A negative nitrite result combines with a positive leukocyte esterase result is cause for further diagnostic tests (urine culture, dip slide, or sediment). This is because previous research has shown the nitrite test to be specific but non-sensitive for UTI, corroborating the results we have found in our study. The inverse is true for the leukocyte esterase test, which is sensitive but non-specific [22–25]. Results of our research underline once again that the urinary dipstick on its own is not accurate enough to predict UTI reliably, but that GPs should consider other factors such as patient symptoms [23, 26–28]. Currently, GPs in Norway and Sweden do not routinely employ the urinary dipstick. For acute cystitis, Norwegian and Swedish GPs base their diagnosis on patient symptoms alone and the urinary dipstick is only used in severe cases [13,14]. However, Bollestad et al. suggested to reconsider the current way of diagnosing UTI, since they showed that symptom severity did not correlate with bacteriuria or symptom duration [29].

## Conclusion

Both visual and automated dipstick analyses provide fast results when performed complying with the instructions for use. Automated and visual dipstick analysis perform similarly in predicting bacteriuria when read and interpreted by researchers in a central laboratory. However, automated analysis requires maintenance and occasionally measurement errors can occur.

## Ethical approval

The medical ethical board of the Maastricht University Medical Centre + approved this study and decided that it is not subjected to the Dutch Medical Research with Human Subjects Law (ref METC 2022-3363).

## Appendix 1. Comparison of visual and automated interpretation of the nitrite test

**Table:**

Automated visual	Negative	Positive
Negative	192	18
Positive	6	86
		95% CI
Cohen’s κ	0.82	(0.81–0.83)

CI = confidence interval.

## Appendix 2. Comparison of visual and automated interpretation of the leukocyte esterase test

**Table:**

Automated visual	Negative	Positive
Negative	65	7
Positive	9	221
		95% CI
Cohen’s κ	0.86	(0.80–0.91)

CI = confidence interval.

## Appendix 3. Comparison of visual and automated interpretation of the erythrocyte test

**Table:**

Automated visual	Negative	Positive
Negative	37	47
Positive	3	215
		95% CI
Cohen’s κ	0.51	(0.43–0.58)

CI = confidence interval.

## Appendix 4. Test characteristics of visual and automated interpretation of the nitrite test

**Table:**

Culture visual	Negative	Positive	Culture automated	Negative	Positive
Negative	139	71	Negative	130	68
Positive*	4	88	Positive*	13	91
Test characteristics visual		95%CI	Test characteristics automated		95% CI
Sensitivity	0.55	0.48–0.63	Sensitivity	0.57	0.50–0.65
Specificity	0.97	0.95–1.00	Specificity	0.91	0.86–0.96
PPV	0.96	0.92–1.00	PPV	0.88	0.81–0.94
NPV	0.66	0.60–0.73	NPV	0.66	0.59–0.72

Based on a prevalence of 0.53.

*Positive for nitrite and/or leukocyte esterase.

CI, confidence interval; NPV, negative predictive value; PPV, positive predictive value.

## Appendix 5. Test characteristics of visual and automated interpretation of the combined nitrite and leukocyte esterase test

**Table:**

Culture visual	Negative	Positive	Culture automated	Negative	Positive
Negative	53	19	Negative	59	15
Positive*	90	140	Positive*	84	144
Test characteristics visual		95%CI	Test characteristics automated		95% CI
Sensitivity	0.88	0.83–0.93	Sensitivity	0.91	0.86–0.95
Specificity	0.37	0.29–0.45	Specificity	0.41	0.33–0.49
PPV	0.61	0.55–0.67	PPV	0.63	0.57–0.69
NPV	0.74	0.63–0.84	NPV	0.80	0.71–0.89

Based on a prevalence of 0.53.

*Positive for nitrite and/or leukocyte esterase.

CI, confidence interval; NPV, negative predictive value; PPV, positive predictive value.
